# Controlling Droplet Marangoni Flows to Improve Microscopy-Based TB Diagnosis

**DOI:** 10.3390/diagnostics11112155

**Published:** 2021-11-21

**Authors:** Stephanie I. Pearlman, Eric M. Tang, Yuankai K. Tao, Frederick R. Haselton

**Affiliations:** Department of Biomedical Engineering, Vanderbilt University, Nashville, TN 37235, USA; stephanie.i.pearlman@vanderbilt.edu (S.I.P.); eric.m.tang@vanderbilt.edu (E.M.T.); yuankai.tao@vanderbilt.edu (Y.K.T.)

**Keywords:** tuberculosis, sputum smear, microscopy, point-of-care diagnosis, low-resource, Marangoni flow, coffee ring, nanoparticle, Ziehl–Neelsen, acid-fast

## Abstract

In developing countries, the most common diagnostic method for tuberculosis (TB) is microscopic examination sputum smears. Current assessment requires time-intensive inspection across the microscope slide area, and this contributes to its poor diagnostic sensitivity of ≈50%. Spatially concentrating TB bacteria in a smaller area is one potential approach to improve visual detection and potentially increase sensitivity. We hypothesized that a combination of magnetic concentration and induced droplet Marangoni flow would spatially concentrate *Mycobacterium tuberculosis* on the slide surface by preferential deposition of beads and TB–bead complexes in the center of an evaporating droplet. To this end, slide substrate and droplet solvent thermal conductivities and solvent surface tension, variables known to impact microfluidic flow patterns in evaporating droplets, were varied to select the most appropriate slide surface coating. Optimization in a model system used goniometry, optical coherence tomography, and microscope images of the final deposition pattern to observe the droplet flows and maximize central deposition of 1 μm fluorescent polystyrene particles and 200 nm nanoparticles (NPs) in 2 μL droplets. Rain-X^®^ polysiloxane glass coating was identified as the best substrate material, with a PBS-Tween droplet solvent. The use of smaller, 200 nm magnetic NPs instead of larger 1 μm beads allowed for bright field imaging of bacteria. Using these optimized components, we compared standard smear methods to the Marangoni-based spatial concentration system, which was paired with magnetic enrichment using iron oxide NPs, isolating *M. bovis* BCG (BCG) from samples containing 0 and 10^3^ to 10^6^ bacilli/mL. Compared to standard smear preparation, paired analysis demonstrated a combined volumetric and spatial sample enrichment of 100-fold. With further refinement, this magnetic/Marangoni flow concentration approach is expected to improve whole-pathogen microscopy-based diagnosis of TB and other infectious diseases.

## 1. Introduction

As the leading cause of infectious disease death worldwide, the global burden of tuberculosis (TB) disease today stands at 10 million cases per annum, with 12% of individuals dying from TB disease. The causative bacterial agent of TB, *Mycobacterium tuberculosis* (*M.tb*), is also the most common opportunistic infection and leading cause of death in HIV-infected persons in developing nations [1]. The inability to rapidly and accurately diagnose TB disease and evaluate resistance with high sensitivity and specificity at the point-of-care (POC) continues to be a confounder in disease transmission, patient mortality and morbidity, and time to treatment initiation in the developing world; this is a major barrier to effective control of TB.

In 2012, the World Health Organization endorsed the nucleic acid amplification-based GeneXpert MTB/RIF assay as a frontline diagnostic and drug resistance test for TB in endemic settings [2]. Unfortunately, reliance on a special instrument possesses significant limitations that hinder its capacity to serve as a comprehensive screening tool. Most crucially, for example, in South Africa, where Xpert is widely used, Xpert devices are commonly located in centralized tertiary healthcare facilities in densely populated areas [3]. This leaves individuals in rural, sparsely populated areas without immediate access to Xpert testing, requiring patient specimens be shipped from remote clinics to testing centers, imposing a lengthy turnaround time (days to weeks) for what should be a two-hour diagnostic test. This exacerbates pre-treatment loss to follow-up rates, which can be up to 38% for patients diagnosed with TB in both rural and urban pats of the developing world [4]. In addition, Xpert cartridge stock-outs and supply chain mismanagement, interruptions in electricity, unpredictable transport of patient specimens from off-site locations, and high start-up costs have impeded implementation of Xpert [5]. Knowing these barriers, researchers have shown that case detection has not significantly improved with the rollout of Xpert, and that the number of patients lost to follow-up did not decrease following TB diagnosis with Xpert [5,6,7]. Thus, empiric treatment without molecular diagnosis remains commonplace [4].

In the absence of any reliable biomarker-based POC diagnostic test for TB, the gold standards for accurate TB diagnosis continue to rely on microscopic pathogen detection paired with diagnostic confirmation using bacterial culture methods [1]. Sputum smear staining and microscopy has the advantages of having high specificity (≈100%) while being inexpensive and easy to perform. Unfortunately, these advantages do not overcome poor diagnostic sensitivity (≈50%), subjectivity, and laboriousness. Manual inspection of stained sputum smear slides requires visual inspection of a minimum 100 fields of view using high-power magnification, which can take up to 3 h, depending on the degree of infection and the microscopist experience [8]. Understaffed and busy clinics that continue to rely on smear inspection are especially prone to false negatives resulting from improper examination [9].

Spatial concentration of TB bacteria has the potential to reduce inspection time of patient samples, which could be further improved when paired with TB-specific volumetric concentration strategies. Using what is known about the hydrodynamics of an evaporating drop, spatial concentration is theoretically achievable without the need for specialized equipment. Our group and others have extensively studied the flow and deposition patterns of particles in drying drops and have shown that these microfluidic properties can achieve spatial concentration at the edge or center of the drop [10,11,12,13].

Our group has previously published two biosensor designs that make use of the radial flows found in evaporating sessile droplets [11,13]. The first design used the primary radial flow to deposit polystyrene particles at the edge of an evaporating drop, creating what is colloquially known as a “coffee ring” [13]. Our second published design used secondary radial flows, known as a Marangoni flows, to concentrate particles in the center of a drying drop. This design used an antibody recognition system to induce particle aggregation in the presence of biomarker, followed by deposition in the center of the evaporating drop [11]. A larger deposition pattern in the center of the drop corresponded to a greater biomarker quantity, and this could be quantitatively measured. The assay also achieved a limit-of-detection (LOD) of picomolar biomarker concentrations. The system included glycerol to remove interference of salt crystallization and enhance the Marangoni flows. It also used a polydimethylsiloxane (PDMS) substrate instead of glass, which has the required material properties for production of Marangoni flows radially inward along the substrate. Unfortunately, both the substrate and solvent material properties that made this assay work are not compatible with acid-fast staining protocols used at the POC. In addition, both assays use 1 μL drops, a volume too small for suspension of a large-volume sample after volumetric concentration.

**Figure 1:**
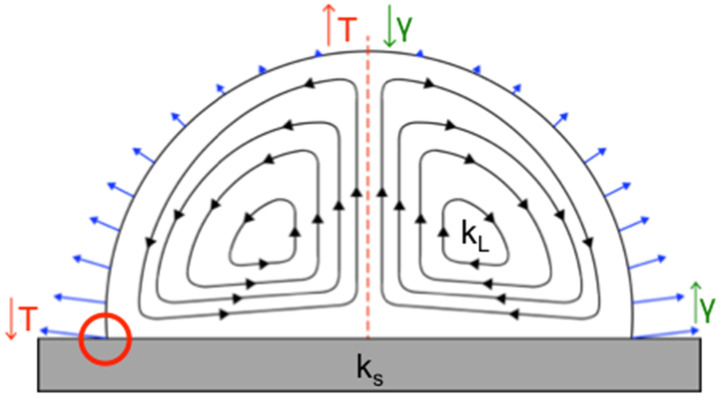
Physical phenomena that contribute to Marangoni flow formation and direction. Surface tension (γ) and thermal conductivity (k) of the surface (k_s_) and droplet solvent (k_L_) can be manipulated to create inward- or outward-oriented Marangoni flow patterns in an evaporating droplet. In this case, nonuniform evaporation along the liquid–air interface of the droplet (blue triangle arrows) occurs. This creates a temperature gradient (T) that results in a nonuniform surface tension gradient. When k_s_ < 1.45 k_L_, where the temperature at the top of the droplet is warmest, while the contact line is coldest (indicated by red circle), the microfluidic flows within the droplet circulate radially inward along the solid substrate (black triangle arrows) independent of surface tension [10,11] (Adapted from ref. [11]).

**Figure 2:**
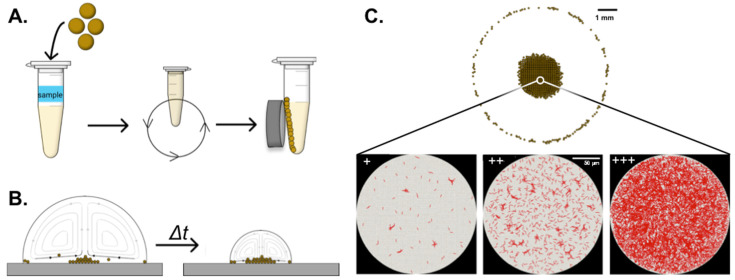
Workflow for combined volumetric and spatial concentration of *M.tb*. (**A**) Magnetic nanoparticles are added, incubated with the sample to capture bacilli, and then magnetically separated from the sample. After washing, the bound sample and nanoparticles are recovered in 50 μL PBS-T. (**B**) The recovered sample is deposited on the Rain-X^®^ + poly-L-lysine-coated microscope slide and evaporated at room temperature over a period of time (Δt). (**C**) The dried sample is stained using the Ziehl–Neelson method, and the central Marangoni deposition area is inspected using microscopy at 1000× magnification. Representative positive samples with increasing *M.tb* concentrations are shown. (+)—10^4^ bacilli/mL; (++) 10^5^ bacilli/mL; (+++) 10^6^ bacilli/mL. Black scale bar—1 mm; white scale bar—50 μm.

Here, we describe a different design for spatial concentration of *M.tb* bacilli for use in microscopic sputum smear inspection using Marangoni flow (Figure 1). In this report, we detail the design and testing of potential materials before comparing our method to direct sputum smear. Figure 2 demonstrates combined magnetic enrichment and spatial concentration using nanoparticles (NPs) and Marangoni flows, achieving a simple workflow for use in low-resource settings.

## 2. Materials and Methods

### 2.1. Mycobacterial Strains, Culture Conditions, and Electroporation

*Mycobacterium bovis* Karlson and Lessell TMC 1011 (BCG Pasteur) (ATCC^®^ 35734^TM^, Manassas, VA, USA), referenced here as *M. bovis* BCG (BCG), and *Mycobacterium smegmatis* (Trevisan) Lehmann and Neumann mc(2)155 (ATCC^®^ 700084^TM^), referenced here as *M. smegmatis* (*M. smeg*), were cultured in BD Difco^TM^ Middlebrook 7H9 Broth (FisherSci, Hampton, NH, USA, Cat# DF0713-17-9) supplemented with 10% BD BBL^TM^ Middlebrook OADC Enrichment (FisherSci, Cat# B12351) and 0.05% glycerol, or on BD Difco^TM^ Middlebrook 7H10 agar (FisherSci, Cat# DF0627-17-4) supplemented with OADC and glycerol. All BCG cultures were grown at 37 °C with constant shaking at ≈200 rpm, and *M. smeg* cultures grown at 20 °C with constant shaking at ≈200 rpm. *M. smeg* was cultured at room temperature as a preventative measure to reduce contamination potential of the incubator and BCG cultures with the faster growing mycobacterial strain. Following electroporation, outlined below, transformed *M. smeg* were grown using the indicated media with 100 μg/mL Hygromycin B (RPI Corp., Mt. Prospect, IL, USA, H75020) supplement to maintain selection of bacteria expressing the plasmid.

Plasmid pMyCA-mCherry was a gift from Young-Hwa Song (Addgene, Watertown, MA, USA, plasmid #84272; http://n2t.net/addgene:84272; RRID: Addgene_84272; last accessed 18 November 2021) [14]. This plasmid was used to create fluorescent *M. smeg* bacilli for imaging. The plasmid arrived as a bacterial stab of transformed *Escherichia coli* DH5α, which were expanded into 100 mL cultures in LB (Luria Broth) liquid culture medium (RPI Corp., L24040) containing 100 μg/mL Hygromycin B at 37 °C with shaking at 200 rpm. The plasmid was isolated from the cultures using the QIAprep^®^ Spin Miniprep Kit (Qiagen, Hilden, Germany, Cat# 27104).

*M. smeg* was electroporated as indicated by Goude and Parish [15], substituting the culture media above into the *M. smeg* electroporation protocol. Following electroporation and plating on selection agar, *M. smeg* was cultured at 37 °C for three to five days, before transformed colonies were transferred into liquid culture. *M. smeg* electroporated with water instead of plasmid DNA did not grow on the Hygromycin B selection plates. Pink colonies only grew on the selection plates that were electroporated with plasmid DNA.

### 2.2. Microscope Slide Coating with Candidate Substrate Materials

Fisherbrand^TM^ Premium Plain Glass Microscope Slides (FisherSci 12-544-1) or Fisherbrand^TM^ ProbeOn^TM^ Slides (FisherSci, 15-188-51) were cleaned using 70% ethanol/30% 1N HCl, rinsed generously with MilliQ water, and dried at room temperature before use. Indium tin oxide (ITO) glass (MilliporeSigma, Burlington, MA, USA, 703192-10PAK) was untreated and used directly from packaging. Krayden Dow Corning Sylgard 184 Silicone Elastomer Kit (FisherSci, NC9285739), referred to here as PDMS, was prepared using a 10:1 Base Polymer (Part A) to Curing Agent (Part B) ratio, poured into a flat aluminum foil-lined tray, degassed under vacuum, and placed at 65 °C overnight to cure. PDMS was wiped clean with 70% ethanol before use to ensure any residual contaminates from handling were removed. The thermal conductivities of each candidate substrate can be found in Table 1.

All coatings were applied to plain glass microscope slides after washing. Rain-X^®^ Original Glass Water Repellent (US# 800002242) and Rain-X^®^ Interior Glass Anti-Fog^TM^ (US# 21106DW) are polysiloxane coatings that covalently modify glass surfaces to which they are applied [16,17]. Rain-X^®^ coatings were poured onto a clean microfiber cloth and applied to the surface of the microscope slide in a circular motion three times, allowing the coating to dry between coats. Residual Rain-X^®^ was removed using a clean, dry microfiber cloth. WD-40^®^ Specialist^®^ Dirt and Dust Resistant Dry Lube PTFE Spray (WD-40, 300059) was applied by spraying onto the surface of an upright microscope slide for 1 to 2 s, allowing excess liquid to drip down the slide onto a Kimwipe^®^. With the excess removed, the slides were placed horizontally to finish drying. DuPont^®^ Non-Stick Dry Film Lubricant with Teflon^®^ fluoropolymer (DNS040101) and Sigmacote^®^ (SigmaAldrich, St. Louis, MO, USA, SL2-100ML), a glass siliconizing agent, were poured into a 50 mL conical, and microscope slides were submerged upright for 1–2 s, and then removed. Excess liquid was allowed to drip off the end of the upright microscope slide onto a Kimwipe^®^. With the excess removed, the slides were placed horizontally to finish drying.

**Table 1:** Thermal conductivities of candidate substrate materials.

Material	Thermal Conductivity (W m^−1^ K^−1^)
Polysiloxanes *	0.077–0.150 [18,19]
Sylgard 184 polydimethylsiloxane (PDMS)	0.27 [20]
Polytetrafluoroethylene (PTFE)	0.245 [21]
Borosilicate glass	1.21 [22]
Indium tin oxide (ITO)	10.2 [23]

* Exact composition of Sigmacote^®^ and Rain-X^®^ [16,17] polysiloxane coatings unavailable from manufacturers.

### 2.3. Contact Angle Measurements of Candidate Substrate Materials

Contact angle of MilliQ water was measured on each substrate material using a ramé-hart Model 200-F4Goniometer (Succasunna, NJ, USA) and DROPimage Advanced Software v2.6.1. The instrument was used as per the manufacturer’s instructions for measurement of contact angles on a level surface (CA Tools > Contact Angle) and calibrated using the 4 mm ball before use. The baseline setup for the tilt of the substrate had no more than ±0.02% difference between the left and right intercepts for all droplets measured. A minimum of 13 (n ≥ 13) 2 μL MilliQ water droplets were measured for each substrate.

### 2.4. Droplet Deposition Pattern Screening of Candidate Substrates

To assess the final deposition of particles in a drying droplet on each substrate, we prepared solutions containing either 1 μm yellow-green fluorescent polystyrene particles (PS) (ThermoFisher, Waltham, MA, USA, Cat# F8768, Lot# 1878394) at a concentration of 10^6^ particles/μL, or 200 nm streptavidin-coated magnetic NPs (OceanNanotech, San Diego, CA, USA, SV0200) at 0.5 μg/μL, in 0.5× PBS + 0.01% Tween-20 + 8% glycerol [11]. Two microliter droplets were deposited in triplicate on each substrate and allowed to dry while being covered with a box to prevent environmental airflow variations during evaporation. Droplets were imaged on a Nikon TE-200U microscope using a 2x objective and DS-U3 Color Camera in Nikon NIS Elements AR 4.13. NP droplets were measured using white light. The fluorescent polystyrene particles were imaged using a fluorescent bulb and filter cube with FITC Ex/Em filters.

### 2.5. Droplet Deposition Pattern Screening with Decreasing Concentration of Glycerol

Using the same particle concentrations listed, we made 0.5× PBS + 0.01% Tween-20 (PBS-T) [11] with either 0%, 2%, 4%, and 8% glycerol, wherein 2 μL droplets were deposited on Rain-X^®^ Original Glass Water Repellent-coated microscope slides in triplicate and were allowed to dry while covered, and were imaged as previously indicated. In addition, the same protocol was repeated with mCherry *M. smegmatis* (*M. smeg*) at a concentration of 10^6^ bacteria/μL (estimated using absorbance at 600 nm), being washed once in 1× PBS before suspension in the droplet solutions. The *M. smeg* evaporated droplets were imaged using a Texas Red Ex/Em filter and were imaged using a 4× objective instead of 2× to improve visualization of the bacteria deposition in the droplet given their small size and lower fluorescence, relative to the PS particles.

### 2.6. Imaging and Analysis of Marangoni Flow Fields in Evaporating Droplets Using Optical Coherence Tomography (OCT)

Droplet flow imaging was performed using a custom spectral-domain optical coherence tomography (OCT) system. OCT is an interferometric imaging technique that allows for the acquisition of a single line in depth for each laser beam position on the sample [24]. By raster scanning the laser beam using mirrors, 2D cross-sectional and 3D volumetric images can be rapidly constructed. A superluminescent diode (Superlum, Carrigtwohill, Cork, Ireland) centered at a wavelength of 889 nm with a 93 nm bandwidth was used for illumination. The resolution of the system was measured to be 4 µm in the lateral dimension and 2 µm in the axial dimension. The imaging system was tilted slightly to reduce the effects of specular reflection during manual alignment to the diameter of a 1 µL droplet. Backscattered light was detected using a CMOS camera (Basler Sprint, spL4096) running at a line rate of 45 kHz. Cross-sectional images were acquired with 2048 pixels in depth and 2000 lines per frame over a 5 mm field-of-view. Due to improvements in hardware and software, the temporal sampling rate was increased from the method reported in Trantum et al. [12]. A total of 800 repeated frames were taken at the same position for a total acquisition time of 36 s per measurement in order to monitor the changes in particle flow and droplet evaporation. For each droplet, measurements were taken at two-minute intervals beginning at one minute following droplet placement until complete evaporation.

Raw OCT spectral data were converted to tag image files (TIFF) using conventional post-processing techniques that include linearizing the spectrum with respect to wavenumber, performing dispersion compensation to reduce axial distortions, and finally converting wavenumber to depth using the Fourier transform. TIFF images were then imported into ImageJ for analysis. Time-lapse composites were created for each measurement by averaging the corresponding 800 frames acquired at the same position and adjusting contrast to better visualize particle flow.

### 2.7. Preparation and Spot Size Assessment of Rain-X^®^-Coated Slides with Poly-L-lysine Layer for Improved Heat Fixation of Magnetic Nanoparticles

**Figure 3:**
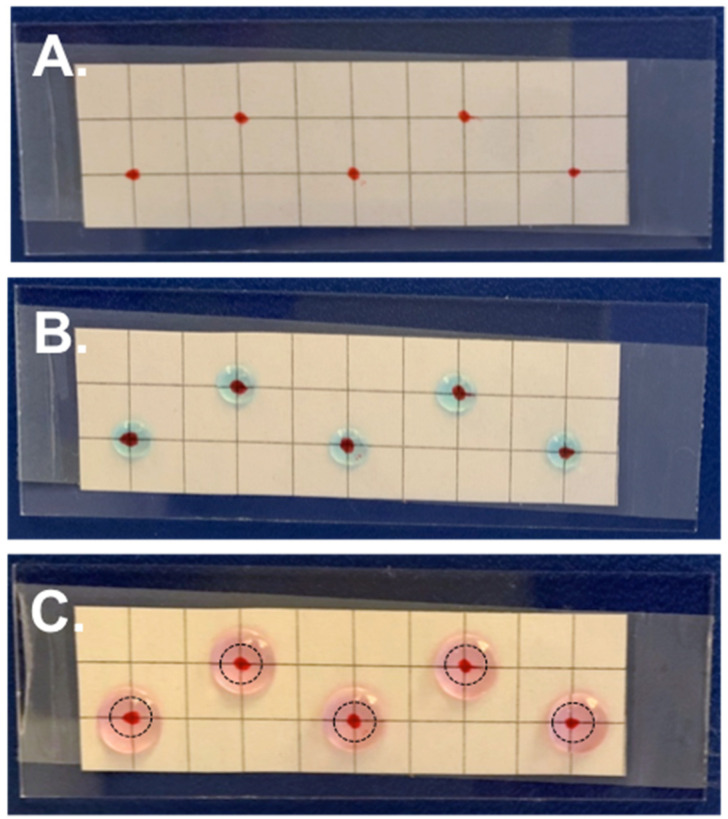
Deposition of poly-L-lysine onto Rain-X^®^-coated slide. (**A**) A marked grid was affixed to the back of the microscope slide using tape. (**B**) Droplets 10 μL in size containing 0.01% poly-L-lysine were spotted onto the microscope slide where indicated. (**C**) After removing excess poly-L-lysine, 50 μL droplets containing the volumetrically concentrated samples were placed over the poly-L-lysine landing zone and allowed to dry. Location of landing zone in center of droplets indicated by dashed circle. Dyes added to droplets for visualization.

Water Repellent Rain-X^®^ was applied to microscope slides as indicated. A grid was drawn onto paper and attached to the back of the microscope slide using transparent tape. Where indicated by the grid, droplets of 1–10 μL containing 0.01% poly-L-lysine (PLL) (MilliporeSigma, P8920-100ML) were applied and allowed to rest for 5–10 min (Figure 3). For a negative control, 10 μL of MilliQ water was placed on the grid in place of PLL. Excess PLL solution was carefully removed through capillary action using a dry Kimwipe^®^, and the slides were baked at 65 °C for one hour and stored in a closed container at room temperature until use. The positive charge of the PLL polymer helps enhance electrostatic interactions between the positively charged surface and negative charges of in the sample, usually located on cell or tissue membranes. See the Results for further discussion.

After, a solution of 0.2 μg/μL of 200 nm streptavidin-coated magnetic NPs was prepared, and 50 μL droplets were deposited over the grid (Figure 3). The droplet was positioned such that the center of it was centered over the applied PLL spot coating. Drops were allowed to evaporate uncovered at room temperature until dry. Bright field microscope images of the dried spots were taken at 1× magnification. Particle deposition area in the center of each droplet was measured by drawing a “polygon” around the outer edge in the raw image in NIS Elements.

### 2.8. Preparation of Pure Mycobacterial Samples

Mycobacteria have a propensity to clump in culture, and it is necessary to dissociate these clumps to obtain a primarily unicellular suspension. Suspension mycobacterial cultures were collected and centrifuged in 50 mL volumes at 1200 rcf for 5 min. The supernatant was removed, and 10 glass beads, 3 mm in size, were added to the cells, which were then shaken vigorously for 1 min. After allowing the aerosols to settle, 6 mL of 1× PBS were added to the cells. The sample was rocked back and forth to wash the walls of the tube, and then allowed to sit for 5 min. After, the upper 5 mL of cell suspension was removed and filtered through a 70 μm cell strainer (FisherSci, Cat# 22363548). New glass beads were added to the cell solution and shaken a second time for 1 min. After shaking, the cell solution was allowed to stand for 10 min, and the upper 4 mL suspension was recovered and used for experiments.

Optical density measurements at 600 nm (OD600) were taken using a Go Direct^®^ SpectroVis^®^ Plus Spectrophotometer (Vernier, Beaverton, OR, USA GDX-SVISPL) and were used to estimate the number of cells/mL. Because of the difficulty accurately counting bacterial cells, cell solutions at various optical density measurements were counted on a BD FACSAria III flow cytometer (Franklin Lakes, NJ, USA) alongside a known quantity of Countbright^TM^ Absolute Counting Beads (ThermoFisher, C36950). It was assumed that one event corresponded to one bacterial cell, and a standard curve correlating Flow Cytometry Events vs. OD600 was created. This was used to estimate the number of cells in a suspension before each experiment (Figure S1). A 10-fold dilution series of bacteria containing 10^6^ bacilli/mL down to 10^3^ bacilli/mL was created, and direct smears of the dilution series were created to determine that the estimated number of bacteria corresponded to what was present on the slide before use.

### 2.9. Processing of Pure Mycobacterial Samples

All stains and buffers used were 0.22 μm sterile filtered before use. Each 2.020 mL sample was split into three parts for a paired comparison. First, 20 μL was removed and applied to a 2 cm^2^ area of a plain glass microscope slide treated with 0.01% poly-L-lysine, creating a direct smear, or unprocessed control. This is the same volume of sputum that is sampled, smeared, and stained for diagnosis [25]. The remainder of the sample was split into two individual 1 mL fractions. Both fractions underwent the same volumetric concentration protocol. The volumetric only control sample was recovered into a final volume of 20 μL and applied to a 2 cm^2^ area of a plain microscope slide. A 20 μL volume was chosen as the volumetric-only control resuspension volume because it is the same volume of liquid used in a direct smear.

The second sample, which used combined volumetric concentration + Marangoni enrichment, was recovered into a final volume of 50 μL, and had the entire 50 μL of the recovered sample placed onto a Water Repellent Rain-X^®^ treated slide with 10 μL spots of PLL; droplets were placed on the grid indicating where the PLL spots were located, as described previously, and allowed to dry completely at room temperature in a closed biosafety hood, with airflow turned off. All samples were placed on their own microscope slide to ensure that no contamination occurred between samples. With all samples dry, slides were heated on a heat block at 65 °C for 2 h to completely inactivate and heat fix the BCG onto the slide, followed by Ziehl–Neelsen staining using a BD BBL/Difco TB ZN Stain Kit, according to the manufacturer’s directions (FisherSci, Cat# B12520). A piece of blotting paper (GE Lifesciences, Marlborough, MA, USA, Cat# 3030-861) was used to prevent the sample from drying out while heating the sample with the first carbol fuschin stain. All samples were glycerol mounted in 90% glycerol, 10% 10× PBS under a #0 cover glass (Thor Labs, Newton, NJ, USA, CG00K), and sealed with nail polish in preparation for imaging.

For the samples that underwent volumetric processing, 10 μL of 200 μm streptavidin NPs (OceanNanotech, SV0200) were added to the BCG-containing samples, and the mixture was briefly vortexed and then placed on a lab rotisserie for 1 h. These sized nanoparticles were selected because individually they are smaller than the diffraction limit of light and would not obscure bright field visualization of bacteria. Samples contained 10-fold dilutions of BCG bacteria as specified. After binding, samples were magnetically separated on a magnetic tube rack for a minimum of 30 min. The unbound supernatant was removed, and the samples were washed twice with 1 mL of 1× PBS + 0.01% Tween-20. Finally, volumetrically concentrated samples were suspended in 0.5× PBS + 0.01% Tween-20 (PBS-T), as previously indicated; placed onto the Rain-X^®^ + PLL prepared slide; and allowed to dry overnight in a closed biosafety cabinet with the airflow turned off (Figure 2).

### 2.10. Microscope Image Post-Processing

Images were altered post-collection to improve visual appearance for publication as follows. To reduce the effects of uneven illumination using ImageJ [26], we subtracted a representative background image from the original deposition image for NP droplets. All images had brightness and contrast adjusted as needed to improve image visualization/printing for publication. Differences in the “Auto White” setting of NIS Elements during image collection resulted in varied color of the PS particle images; for this reason, all PS fluorescent images were recolored green in Microsoft PowerPoint for consistency in the final manuscript image. No specific quantitative data were taken from any of the images pre- or post-processing.

### 2.11. Imaging of Paired Processing Methods

BCG processed samples were imaged on an Olympus CX23. This is a model of microscope that is commonly available in primary rural clinics in low-resource areas of the world (W. Silva, personal communication, 19 March 2019) [27], and this is why it was chosen for final sample imaging in place of the Nikon TE-200U. Samples were manually inspected at 1000× total magnification under oil immersion and assessed by inspecting up to 100 fields of view and recording the number of acid-fast bacilli visualized using the semi-quantitative method outlined in the Global Edition of the Laboratory Diagnosis of Tuberculosis by Sputum Microscopy Handbook [28]. Cells had to be stained a dark pink or red to be considered “positive” for a BCG cell. Poorly stained cells or contaminants were not counted towards the cell count, ensuring conservative estimates. Images were taken using a Gotsky Microscope Lens Adapter with WF 10× Eyepiece with 30 mm Tube Smartphone Camera Adapter (Amazon.com, https://www.amazon.com/gp/product/B085C6ZKSL/ref=ppx_yo_dt_b_asin_title_o00_s00?ie=UTF8&psc=1, last accessed 26 April 2021) and an iPhone XR, Model MT3L2LL/A. All samples were imaged at 1000× under oil immersion, and images were selected to be representative of all samples inspected. Top view images of the Marangoni droplets were also taken at 40× total magnification for a global view of the evaporated droplets. A microscope micrometer was used to determine scale.

## 3. Results

There are two types of radial flows observed in an evaporating sessile droplet [29,30,31,32,33,34]. The first, which are found in droplets containing pure water, are capillary flows responsible for the “coffee ring effect,” carrying suspended colloids to the edge of the droplet radially outward along the substrate, resulting in a nonuniform deposition pattern across the droplet area upon complete drying, with particles at the edge of the droplet upon drying [29,30,31]. A secondary radial flow forms when the nonuniform evaporation rate (indicated by blue arrows in Figure 1) of the sessile drop, thermal conductivities of the substrate and droplet solvent, and surface tension of the drop all interact synergistically, resulting in a temperature gradient across the droplet volume. This creates predictable, specific flow patterns, known as Marangoni flows (Figure 1) [10,19]. Marangoni flows can move in the same direction or opposite direction of the fluid stream in the coffee ring effect and can result in particles depositing at the edge or center of the drop, or across the entire liquid–solid interface. On the basis of our prior work, we know that when Marangoni flows move inward along a hydrophobic substrate, a clearly defined central deposition of 1 μm particles occurs, forming what is essentially a reverse coffee ring. In addition, Marangoni flows can also be influenced by a nonuniform surface tension gradient [31].

While there are a variety of biological applications using Marangoni flows in evaporating sessile droplets [11,35,36,37,38], unfortunately previous work does not use materials compatible with many microbiological staining protocols. Knowing this, we sought to use established knowledge to intelligently design a new system compatible with cellular biomarkers. This system can then be paired with a volumetric concentration strategy to further improve the results of the method.

We first focused on identifying a new substrate material. Identifying the substrate required that multiple design requirements be taken into consideration. For acid-fast staining, materials should be optically clear, flame-resistant (will not burn with prolonged flame exposure), nonporous, inexpensive, and readily available. It was also important that we preferentially concentrate the NPs and bacteria into the center of the drying droplet, since this would create a small, well-defined area for inspection under a microscope.

It is known that both the droplet contact angle and the ratio of the substrate, k_s_, and droplet solvent, k_L_, thermal conductivities are important in determining the circulation direction of Marangoni flows (Figure 1) [10] in evaporating droplets. Established theory by Ristenpart et al. states that for the Marangoni flows to move inward along the liquid–solid substrate interface and outward along a drop’s liquid–air interface (referenced from here on as the direction of fluid flow along the substrate) can be achieved by keeping the ratio of the thermal conductivities $k_{s}/k_{L}$ less than 1.45. When the ratio is greater than two, the flow field reverses and instead flows outward along the substrate surface. When a ratio between 1.45 and 2 is achieved, the Marangoni flow direction is dependent on the droplet contact angle [10,32]. The direction of the fluid flow [10] and forces applied to the colloids ultimately impact where colloids deposit, and for this reason it was critically important to maintain fluid flow inward along the substrate. Although the contact angle is irrelevant for Marangoni flow direction when $k_{s}/k_{L}$ is less than 1.45 or greater than 2, it can also impact the final particle deposition pattern [10], and therefore we preserved both the contact angle and Marangoni flow direction in our new design. Logically, glass slides used for sputum smear microscopy, and PDMS, the substrate known to yield the desired Marangoni flow patterns [11], were chosen as candidate substrates. Beyond these, surface coating materials containing PTFE or polysiloxanes were selected for initial testing because of their low thermal conductivity (Table 1) and high heat resistance. ITO-coated glass, which meets all the design criteria, with the exception that its high thermal conductivity would produce outward-oriented flow patterns in aqueous droplets, was selected for comparison to the candidate substrates with lower conductivity.

**Figure 4:**
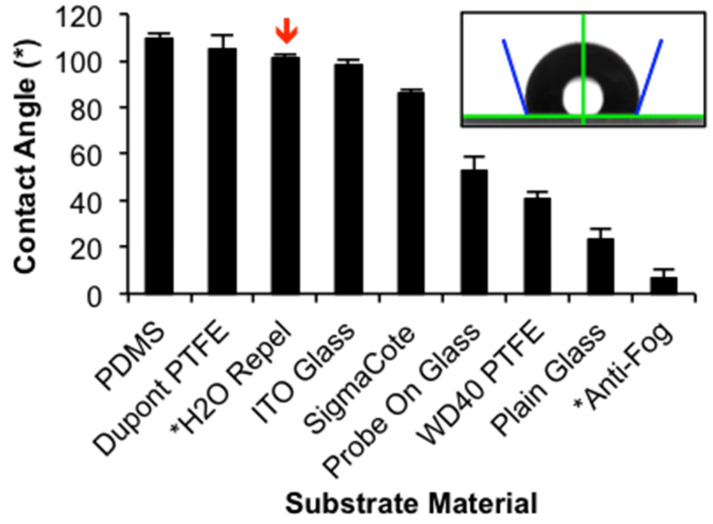
Contact angle measurements, as shown in insert, of 2 μL MilliQ water droplets on each candidate substrate (mean ± s.d.; n ≥ 13). Coatings with a contact angle less than 85° did not centrally deposit particles during evaporation. Red downward arrow indicates substrate selected for final design (H2O Repel). * Indicates Rain-X^®^ product.

We started substrate characterization by measuring the contact angle of water on the substrates. This provided information about how a substrates’ hydrophobicity/hydrophilicity will impact surface tension of the droplet. Figure 4 measured contact angle of 2 μL water droplets on the candidate substrates. The contact angles measured for PDMS (109.6° ± 1.9°), DuPont^®^ PTFE (104.6° ± 6.1°), Water Repellent Rain-X^®^ (101° ± 1.8°), Sigmacote^®^ (86.3° ± 1.2°), plain glass (23.5° ± 4.7°), and Probe On^TM^ glass (53.1° ± 5.6°) are consistent with literature values [39,40,41]. The positive charge of the Probe On^TM^ slides creates a more hydrophobic surface, with polar water molecules being partially repelled by the surface, creating a larger contact angle when compared to a standard glass microscope slide.

When measuring the contact angle for the WD-40^®^ PTFE coating, the dried film readily rehydrated upon drop deposition, and vortices of PTFE coating mixing into the droplets were observed through the side of the droplet on the goniometer during measurement (data not shown). This would explain the smaller contact angle (40.8° ± 2.6°) compared to the DuPont^®^ PTFE coating and reported literature values [41].

The Anti-Fog Rain-X^®^ performed as intended in everyday usage, such as on windshields or glasses, although this was the opposite of the other polysiloxane coatings; by creating a superhydrophilic surface, any water droplets that form on the glass surface are flattened to reduce visual interference. Anti-Fog Rain-X^®^ had the smallest reported contact angle (6.9° ± 3.5°), although within 10 s of droplet deposition, the contact angle was reduced to 0°.

**Figure 5:**
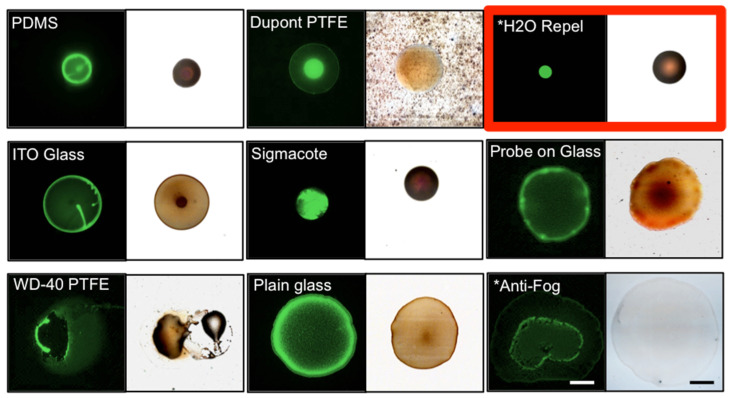
Particle deposition patterns of 2 μL droplets containing green fluorescent polystyrene particles (left of each pair) or 200 nm magnetic nanoparticles (right of each pair) in order of decreasing contact angle. Theory states that Marangoni flows in all droplets are oriented radially inward along the substrate, with the exception of ITO, which is oriented radially outward along the substrate. Because of the strong central deposition pattern, we selected Water Repellent Rain-X^®^ (H2O Repel) to covalently coat microscope slides, creating our solid substrate. Scale bar = 500 μm, n = 3. * Indicates Rain-X^®^ product.

Our goal was to create a highly repeatable, well-defined, small central deposition area that was also easy to find under a microscope, and therefore it was important that we observed central deposition for both nano- and micron-scale particles, colloid sizes expected to be in our final droplet, before moving forward to biological experiments using rod-shaped bacilli. After measuring the contact angle of the selected substrates, we allowed particle-containing drops to evaporate and assessed the deposition pattern of PS particles and magnetic NPs. As shown Figure 5, the materials with contact angles less than 85° failed to concentrate particles in the center of the evaporating droplet. The contact angle is dependent on cohesive and adhesive intermolecular forces within the liquid and between the liquid–solid interfaces. These forces are also at play between the liquid and suspended colloids. These can influence particle deposition directly on the basis of the particles’ chemical composition, not only indirectly by changes in the contact angle and surface tension. Furthermore, surface forces and inertial body forces acting on the particles control the particle trajectory within the flow velocity field and their deposition location. The balance of these forces change with the size, density, shape, and surface composition of the particles, as well as the velocity of the fluid flow, which result in different particle deposition patterns upon drying.

Three substrate surfaces in Figure 5 showed deposition in the center of the droplet for both 1 μm and 200 nm colloidal solutions: Water Repellent Rain-X^®^, PDMS, and Sigmacote^®^. Water Repellent Rain-X^®^ was ultimately selected as the final substrate coating for the design for a few reasons. First, Water Repellent Rain-X^®^ covalently modifies the surface of glass without leaving a residue behind, which is a beneficial design for downstream assay packaging; the slides could be prepared by the manufacturer and would be stable for long-term storage. Second, unlike the Rain-X^®^, Sigmacote^®^ left a residue on the slide that could be disturbed during handling. This would make pre-coated slides more difficult to package in a deliverable assay. It also increases the chance that a sample is lost during the staining process after deposition and drying and makes it more difficult for the sample to physically heat fix to the glass. Moreover, simply, Water Repellent Rain-X^®^ is less expensive and very easy to acquire.

ITO-coated glass demonstrates how the reverse flow pattern changes the applied forces and impact on colloidal deposition. With a high thermal conductivity, theory states that the Marangoni flows are oriented outward along the substrate [10], not inward as desired. This is regardless of its measured contact angle (98.5° ± 1.9°), which is similar to that of PDMS and other candidate surfaces that have highly efficient central deposition. As shown in Figure 5, PS particles (a similar size to individual bacteria) settle at the edges of the drop, and NPs are deposited across the total drop interface, although with some concentration in the middle of the drop.

With a microscopy-compatible substrate determined, testing was then performed to establish a compatible solvent system. Trantum et al. had demonstrated that glycerol was important, although not necessary, for formation of Marangoni flows. In addition, glycerol was added to prevent salt crystallization that interfered with imaging [11]. Unfortunately, glycerol is difficult to remove or evaporate due to its viscosity and high boiling point, making it incompatible with microscopy staining. Because of the acid-fast staining process, salt crystals are rinsed away, eliminating this problem. As shown in Figure S2 and as observed by Trantum et al., removal of glycerol from the droplet still formed centrally located Marangoni deposition in 2 μL droplets of 1 μm PS particles; 200 nm NPs; and *M. smeg*, a nonpathogenic mycobacterial cousin of *M.tb*. Again, theory states that these flows should be directed radially inward along the substrate.

**Figure 6:**
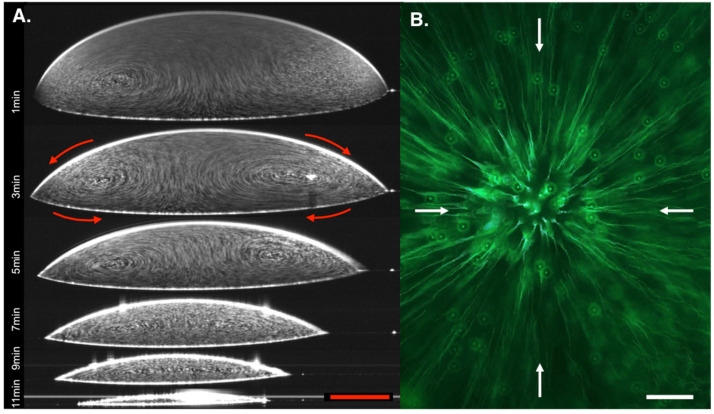
Time-lapse images demonstrate formation of Marangoni flow patterns oriented radially inward along the substrate. (**A**) Starting at 1 min, and every 2 min thereafter until the droplets dried, composite images containing 800 repeated frames in 36 s were taken at the diameter of the 1 μL droplet to image the flow fields produced in the droplet using optical coherence tomography. Flow direction is indicated by red arrows. (**B**) Top-view microscopy image showing movement of particles inward along the substrate surface of a drop in a 5 μL droplet. A single image was captured using fluorescent imaging with an exposure time of 4 s. White arrows indicate flow direction. Sample contains 10^6^ and 10^4^ particles/μL. Composite videos for OCT and microscopy series can be found in the Supplementary Information (Videos S1 and S2). Red scale bar = 125 μm. White scale bar = 100 μm.

After identifying a new substrate material and new solvent system that were both compatible with microscopic staining techniques and met the criteria established by Ristenpart et al., we further confirmed that the final deposition pattern was due to Marangoni flows oriented inward along the substrate using OCT, as we have demonstrated previously [11,12]. Figure 6A shows composite, time-lapse images through the diameter of a 1 μL evaporating droplet containing 10^6^ polystyrene particles/μL. Video S1 uses the same image series to dynamically show that the flow fields are oriented as expected in the inward direction along the substrate, the same direction shown by the black arrows in Figure 1. Reflection off the particles is indicated by the white dots moving within the droplet. During the first minute of evaporation, the microfluidic flows produced by the thermal gradient were initially unstable, possibly due to Bénard–Marangoni instability, though this is just speculative [42]. As time progresses, the chaotic flows stabilized into radially symmetric flows, as seen at 3 min. Due to the small size of the droplet, the accumulation of particles in the center of the droplet was not visible via OCT, but it was clearly observed upon total drop evaporation.

Microscopy was also used to visualize the microfluidic flows. While microscopy cannot image the entirety of droplet flows vertically, the images and videos gathered help to support the data shown in Figure 6A. In Figure 6B, a singular image with a 4 s exposure time was taken near the solvent–substrate interface of a 5 μL droplet containing 10^4^ particles/μL, demonstrating movement radially inward. Video S2 captured a series of images of the same droplet, which dynamically shows the flow field orientation. Images for Video S2 were taken at 1 fps for 40 s.

With promising results in scale experiments and flow confirmation using OCT and microscopy, we performed trials with larger volume droplets containing NPs to confirm that the Marangoni flows occurred independently of droplet size, using the final deposition pattern to assess performance (Figure S3). For droplets 1–100 μL in diameter, all drops exhibited central deposition of the suspended NPs. A 50 μL drop was chosen because it created a small enough central inspection area for efficient inspection without completely obstructing view, whereas smaller droplets resulted in central areas that were too dense to observe bacterial clusters among the NPs. Droplets larger than 50 μL had an extremely long drying time, making it not feasible to perform, given the drying time for a 50 uL droplet already takes a minimum of 5 h. A time series showing the evaporation of a 50 μL drop, demonstrating the formation of the concentrated area during evaporation can be found in Figure S4.

While it seemed the system was ready for use, a critical flaw in the substrate design, which was previously alluded to, became clear and required further design refinement. Because the surface of the microscope slide was covalently modified to become hydrophobic, even with heat fixation, the samples of NPs with and without bacteria were almost completely lost during the staining process; this required testing of a variety of fixation techniques to solve the problem. Specialty microscope slides provided inspiration. Specifically, companies commercially make positively charged slides that aid in sample adherence (such as the Probe-On^TM^ slides used earlier in this study), and at-home recipes for coating slides in poly-L-lysine (PLL) are readily available online. The PLL polymer helps enhance electrostatic interactions between the microscope slide surface and applied sample. We found that coating the entire Rain-X^®^-coated slide in PLL impacted the deposition pattern, leading to uniform deposition across the liquid–solid interface of the drop, likely caused by the positive charge of PLL ionically attracting the bacteria and NPs to the surface with enough force to overcome forces exerted by the radial Marangoni flows.

We then investigated if functionalizing the Rain-X^®^-coated slides in the center of the droplet deposition area with PLL, not the entire slide, could create a “landing zone” for the sample, helping secure it for staining while not significantly changing the deposition pattern. With this, arrays of increasing size PLL spots were placed onto Rain-X^®^-coated slides, followed by NP deposition on top of the array, ensuring the center of the drop was placed over the PLL spot, as indicated by graph paper taped the back of the microscope slide (Figure 3). The grid was removed for staining and imaging, and after analysis of the droplets, we determined that depositing a 10 μL PLL spot in the center of the 50 μL drop deposition area was the best option because it resulted in improved uniformity of the central deposition area (Figure S5). The drawback to using such a large PLL spot is that a small amount of sample does deposit outside the most centrally concentrated area, across other regions of the liquid–solid interface of the drop. However, it was decided that this trade-off is worthwhile, since a larger PLL spot size allows for error as the operator manually deposits the final sample on the microscope slide. The implication is that the most concentrated center created by the Marangoni flows will still land on the PLL area, ensuring the majority of the sample is not lost. Although there are alternative methods to functionally achieve the same thing, such as derivatizing the surface of the microscope slides with antibodies that target both the bacteria or NPs, the protocols to perform this are much more complex, require specialized reagents, and could require specialized storage conditions that in our opinion negate any potential selectivity it would provide.

**Figure 7:**
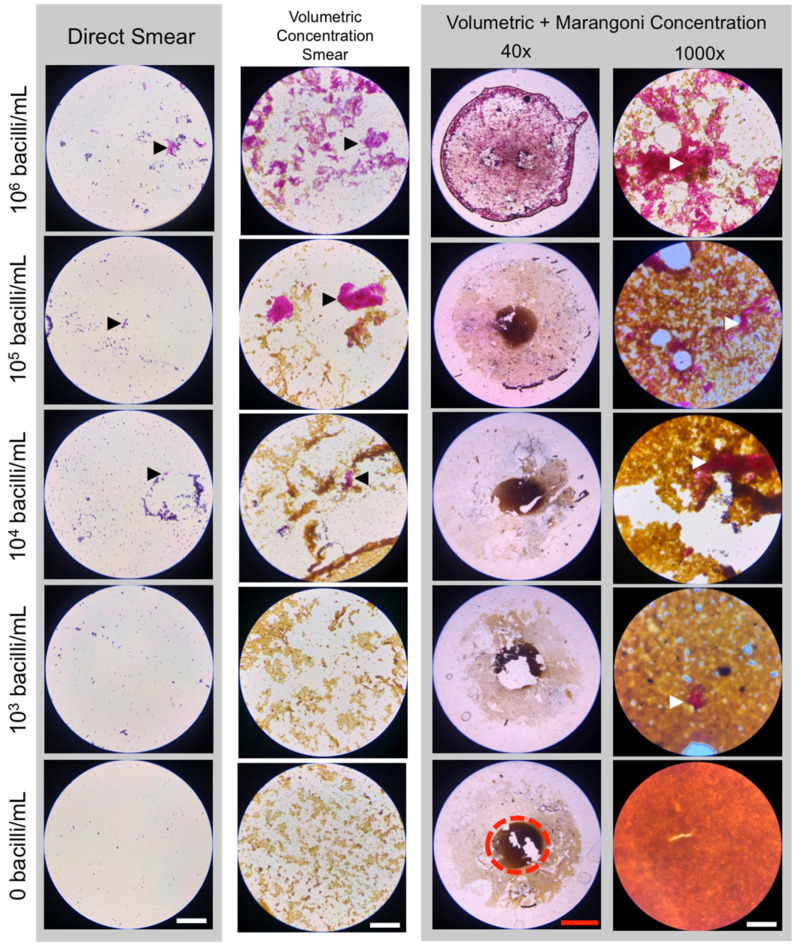
Effect of volumetric concentration and Marangoni spatial enrichment of 50 μL droplets on smear microscopy (n = 3). BCG-stained pink/red with Ziehl–Neelsen stain. Nanoparticles are brown or may appear as red-orange if extremely concentrated, as seen in the lower right most image. Counterstained objects appear dark blue-purple. The most central deposition area inspected for combined volumetric + Marangoni concentration is indicated by a dashed red circle on the 0 bacilli/mL whole drop image at 40× magnification. In the high magnification columns, 1, 2 (black), and 4 (white) arrows indicate a positive smear. Images selected were representative of the samples inspected. White scale bar = 30 μm. Red scale bar = 300 μm.

With the Marangoni flow system finally designed, combined volumetric concentration and spatial enrichment of *M. bovis* BCG was performed. The bacteria were nonspecifically captured on streptavidin-coated NPs and isolated on a stationary magnet. After washing the particles, they were either applied over the standard 2 cm^2^ area, creating a volumetric concentration-only control, or were suspended in 50 μL and applied as a drop to the PLL-Rain-X^®^-functionalized slide. These were both visually and semi-quantitatively compared to a direct, unconcentrated smear (Figure 7). All samples, regardless of processing method, were stained using the gold standard Ziehl–Neelsen staining protocol. Initially, we had designed the method using anti-*M.tb* immunospecific NPs but found that for both naked NPs and isotype control NPs, just as many bacteria were nonspecifically captured by the NPs during volumetric concentration. Those with experience working with mycobacteria know that they are extremely “sticky,” and it can be difficult to prevent nonspecific binding of bacteria to surfaces. It is likely that creating an immunospecific particle will further improve the results shown here.

With the current limit-of-detection for sputum smear microscopy being 10^4^ bacilli/mL [43], as expected, it was generally difficult to find any bacteria on the direct smears for all samples except 10^6^ bacilli/mL. When samples were volumetrically concentrated, higher concentration samples benefitted significantly more than lower concentration samples. Even with volumetric concentration, only 2% of the smear area was inspected, and there were few or no bacteria seen by microscopy in the 10^3^ bacilli/mL and 10^4^ bacilli/mL samples. When volumetric concentration and spatial enrichment were combined, the 10^3^ bacilli/mL and 10^4^ bacilli/mL samples had visible, stained mycobacterial clusters in the central deposition area of the droplet. No bacteria were seen in any of the samples containing 0 bacteria. On a plain glass slide, central deposition of sample NPs did not occur (Figure 5 and Figure S6).

The benefit of combining spatial and volumetric concentration was further seen when samples were semi-quantitatively analyzed using the same examination methods used for diagnostic sputum grading during sputum smear microscopy (Table 2) [28]. Grading of the smears was performed conservatively, given the difficulties of achieving a uniformly single-bacillus suspension with accurate quantification of bacteria in the sample. For the samples with higher concentrations of bacteria, volumetric concentration improved the grading by one grading, but this was not seen in lower concentration samples, particularly the 10^3^ bacilli/mL. Specifically, volumetric concentration on its own failed to improve detection of these samples. With the addition of spatial concentration, these samples all had detectable amounts of bacteria, even with some visible sample loss during staining. Even in areas where bulk sample was lost, stained bacteria were observed, showing that the addition of the PLL spot helped prevent sample loss not just macroscopically but microscopically (data not shown).

## 4. Discussion

Our Marangoni flow-enabled spatial concentration system, combined with volumetric magnetic NP concentration, resulted in a design that outperformed the current technique and was able to visualize bacteria in samples that contained 10× fewer bacteria than the current limit-of-detection for direct sputum smear microscopy (Table 2). This study is the first of its kind to use the microfluidic principles of Marangoni flows to improve microscopic visualization of whole pathogens using standard microscopic staining techniques.

We designed the method with the goal of introducing an improvement to the current workflow, rather than establishing an entirely new diagnostic method. Unlike our previous design, particles that do not aggregate were designed to still deposit in the center of the droplet. We chose this design because it creates a clearly defined, small area for inspection for the presence or absence of *M.tb* bacilli under the microscope. A significant majority of the time and labor that goes into sputum smear microscopic diagnosis of TB is inspection of the smear itself, which is applied over a large 2 cm^2^ area. Concentrating the sample into a smaller known area increases the chances of observing TB bacilli, especially when paired with volumetric concentration. The current smear inspection looks at 100 fields of view, which is equivalent to 2% of the standard smear area. Use of Marangoni concentration deposits the majority of the sample into the most central deposition area (as indicated in Figure 7), increasing the inspected sample area to ≈90%. For a 1 mL sample, volumetric concentration alone into a 20 μL smear concentrates the sample 50×. Spatially, while the sample is primarily concentrated into the center of the droplet, the total area occupied by the sample was 5.3× smaller than the standard smear area, while the most central deposition area was 45× smaller. Combined volumetric and spatial concentration into a 50 μL droplet yielded a sample that was enriched 105-fold. If the method were improved to concentrate 100% of the sample in the most central deposition area of the droplet, perhaps through improvements in the fixation protocol, total enrichment could be up to ≈900×.

To overcome the limitations of bright field microscopy-based inspection of sputum smears, researchers have made a substantial effort to develop fluorescence microscopy (FM) for use with volumetric sample concentration-based methods for TB detection [44,45,46,47,48]. These approaches, centrifugation and magnetic bead separation, unfortunately use time-intensive and nonspecific isolation procedures, increasing labor requirements to the already lengthy inspection protocols. As a consequence, these concentration-based FM smear methods show only modest improvements in clinical sensitivity [49], but reduced specificity [49,50]. In addition, FM inadvertently creates a second barrier for implementation because of its reliance on FM technology that is frequently unavailable in low-resource settings [9]. Other magnetic bead separation strategies use larger particles that obscure visualization of the bacteria, requiring that FM be used for visualization. While these methods have failures that prevent implementation, our design works to overcome the barriers presented. Our method demonstrates the potential for what is achievable with further design of an antigen-specific NP. For the purposes of this study, the “stickiness” of mycobacteria allowed for the use of nonspecific isolation on a simple streptavidin particle, but in practice, other contaminants, such as non-tuberculous bacteria and human cells found in the airways, would also stick to the particles, creating a sample that is impossible to accurately and effectively visualize without providing any specificity. The use of NPs smaller than the diffraction limit of light enabled improved visualization of the sample using bright field microscopy and gold standard acid-fast staining techniques. Although not demonstrated, fluorescent-staining techniques such as auramine O [27] could theoretically be used with our concentration method, but it is not a requirement for implementation such as with prior methods. The newly developed DMN-tre stain would provide the added benefit of not subjecting the sample to repeated harsh chemicals as occurs with acid-fast staining techniques; this would help resolve the issue of sample loss, but fluorescence microscopy would be required for visualization [51].

There are two core phenomena that are important elements of our method. First, during volumetric concentration, the capture of bacteria on NPs form aggregates that are clearly visualized upon staining. Second, thermocapillary Marangoni flows oriented inward along the substrate are important for ensuring central deposition of captured bacteria and NPs. The formation of bacterial aggregates aid in the final visualization of the bacteria in the center of the drop, which are seen as large, three-dimensional pink or red networks of bacteria amongst the NPs (Figure 7). Regardless of the presence or absence of bacteria, the NPs settle in the center of the droplet during evaporation, creating a clearly defined area for inspection.

This study was designed to use known principles of Marangoni flow formation to engineer a new design for microscopic application. Changes from our prior work in both the substrate and solvent were determined. The solutions tested were based on the work of Ristenpart et al. [10] and our prior work [11,12,13]. Having clearly defined goals and design constraints allowed us to focus materials testing while also easily identifying inexpensive solutions for these improvements. The knowledge that Marangoni flows are not formed in pure water [52,53] helped us in modifying the droplet solvent, allowing us to remove what was incompatible with microscopy while maintaining a system that created the desired flow pattern. The addition of the PLL droplet array was a critical design requirement for making our chosen substrate and solvent system useable.

The use of inexpensive, commonly available PLL and Rain-X^®^ for the microscope slide substrate means the cost of the microscope slide is nominally more expensive. Moreover, the cost of the magnetic NPs was only USD 0.56/test at time of publication. Even with the addition of an antigen recognition system to the NPs, this assay could easily be produced at <USD 1.00/test, in addition to the cost of staining reagents already used at the POC. In addition, because Rain-X^®^ covalently modifies the surface of the microscope slide, and PLL-coated slides have a year-long shelf life if stored in a closed container, the slides could ship to decentralized testing sites prepared and ready for use with no required preparation. While PLL spots were applied by hand in this study, automated processing could create highly repeatable and reproducible microscope slides.

This report was designed to be a “proof-of-principle” study, and some limitations remain. As mentioned, it is important that the NPs be immunospecific in the final application, because as prior work demonstrates, nonspecific volumetric concentration of samples does not significantly improve sensitivity and also reduces specificity [49,50]. The use of nonspecific isolation techniques in prior studies and the continued issues seen here demonstrate the difficulty in creating an immunospecific particle for specific capture of mycobacteria. The gap that remains by not solving this problem is not lost, since failing to specifically isolate mycobacteria from a sputum sample will make it impossible to visualize bacteria. Specifically, it is extremely common for other biological contaminants to significantly outnumber TB bacteria in sputum samples; isolation of all biological contaminants will result in a dark blue stain that cannot be accurately read by the microscopist. For methods such as this to work, it is imperative that more development focus on methods to rapidly and specifically isolate mycobacteria. Even better, design and use of an antigen recognition element that can discern between tuberculous and nontuberculous infections would create a diagnostic that satisfies another diagnostic need that is currently insufficiently met using culture [54].

Second, magnetic NPs were chosen specifically because they are smaller than the theoretical diffraction limit of light and are unable to individually obscure bacilli visualization such as with larger magnetic particles. However, together, if spatially concentrated enough, they can obscure imaging. The particle volume used in this study was chosen to yield efficient volumetric concentration while not completely obscuring visualization following spatial enrichment, but in other applications, these parameters would need to be optimized to prevent bulk NP imaging interference.

Third, given this is study intended to demonstrate the improvement of a rapid, POC diagnostic method, it is important that it can be performed in a period no greater than a few hours. In the current iteration, both the time it takes for magnetic separation and the amount of time it takes for the droplet to dry are too long to meet this requirement, and changes to the method design are required. The lower magnetic susceptibility of the NPs, compared to more common ≥1 μm magnetic microparticles used in magnetic bioseparations, significantly extends the time it takes to magnetically separate the sample. When adding the increased viscosity of sputum in a patient sample, the separation using a stationary magnet is not feasible for use at the POC. The use of high-gradient magnetic separation, a technique that has been shown to rapidly and efficiently separate both micro- and NPs from large-volume, high-viscosity samples [55,56], has the potential to solve this problem and should be explored as an alternate to stationary magnetic pooling. This technique may also allow for use of even smaller NPs, providing another solution to bulk NP imaging issues. The use of entire sputum samples, which averages 3 mL in volume [57], can further improve method sensitivity.

To solve the issue of the droplet drying time, the use of multiple smaller droplets instead of a single large droplet can significantly reduce the time to dry, although this could increase the inspection time and/or area. In addition, as demonstrated in microarray development, the use of a greater number of smaller drops rather than fewer larger drops can improve the sensitivity of the method [58]. Further studies should be performed to determine if this theory applies to our application and what the tradeoff between inspection area and sensitivity is. It also may provide a solution to potential NP visualization issues previously mentioned.

## 5. Conclusions

The proposed volumetric magnetic enrichment with Marangoni flow concentration is an effective alternative to the current method of sputum smear microscope-based inspection for TB diagnosis. We have shown that our method outperforms the current gold standard sputum smear method and has advantages over other magnetic bead-based volumetric concentration strategies, namely, the use of smaller particles that do not inhibit bright field imaging. In addition, other methods fail to provide any type of significant spatial concentration after volumetric enrichment. The method is inexpensive, simple, fits within current smear and staining protocols, and does not require any additional specialized equipment that is not already available or easily obtainable and used at the point-of-care where sputum smear inspection is already performed. With further refinement, we expect that this technique can be applied to isolation and microscope-based inspection for other whole pathogens.

## 6. Patents

Vanderbilt University has filed patent applications describing the Marangoni flow-based biosensor assay designs discussed in this publication.

## Figures and Tables

**Table 2:** Semi-quantitative grading for each sample processing method.

		Sample Type		
		Direct Smear	Volumetric Concentration	Volumetric and Marangoni Concentration
Bacteria/mL	10^6^	2+, 2+, 2+	3+, 3+, 3+	3+, 3+, 3+
	10^5^	Sc (4) *, 1+, 1+	2+, 2+, 2+	3+, 3+, 3+
	10^4^	Neg, Sc (7), 1+	1+, 1+, 1+	1+, 1+, 2+
	10^3^	Neg, Neg, Neg	Neg, Neg, Neg	Sc (8), 1+, 1+
	0	Neg, Neg, Neg	Neg, Neg, Neg	Neg, Neg, Neg

* Sc (#) = Scanty (# cells counted), Neg = Negative. See [28] for sputum grading criteria.

## Supplementary Materials

The following are available online at www.mdpi.com/article/10.3390/diagnostics11112155/s1, Figure S1: Flow cytometry curve correlating number of events counted against OD600 of *M. bovis* BCG bacteria. Figure S2: Removing glycerol from droplet solvent did not significantly change particle deposition location in droplets containing 1 μm polystyrene beads, 200 nm iron oxide magnetic nanoparticles, or mCherry-expressing *M. smegmatis* bacteria. Figure S3: The size of the droplet did not impact the final deposition pattern of the droplet, up to a 100 μL volume. Figure S4: Time-lapse imaging of a 50 μL evaporating droplet shows the formation of the central deposition area as the droplet evaporated. Figure S5: Effect of poly-L-lysine landing zone size on area of central Marangoni deposition area. Figure S6: Drying of 50 μL droplet on plain glass did not result in characteristic Marangoni deposition pattern upon complete drying. Supplementary Video S1: Sequential OCT images of evaporating 1 μL droplet containing 106 polystyrene particles/μL, which were 1 μm in diameter. (MP4) Supplementary Video S2: Sequential fluorescence microscopy images at 100× total magnification of evaporating 5 μL droplet containing 104 polystyrene particles/uL, which were 1 μm in diameter (MP4).

## Abbreviations

BCG, *Mycobacterium (M.) bovis* BCG; FM, fluorescence microscopy; ITO, indium tin oxide; LOD, limit-of-detection; *M. smeg*, *Mycobacterium (M.) smegmatis*; *M.tb*, *Mycobacterium (M.) tuberculosis*; NP, nanoparticle; PBS-T, 0.5× PBS + 0.01% Tween-20; PDMS, polydimethylsiloxane; PTFE, polytetrafluoroethylene; PLL, poly-L-lysine; POC, point-of-care; s.d., standard deviation; TB, tuberculosis; ZN, Ziehl–Neelsen.

## References

[1] (2020). Global Tuberculosis Report 2020.

[2] (2011). Policy Statement: Automated Real-Time Nucleic Acid Amplification Technology for Rapid and Simultaneous Detection of Tuberculosis and Rifampicin Resistance: Xpert MTB/RIF System.

[3] (2019). GeneXpert Xpert MTB/RIF National Report March 2019.

[4] MacPherson P., Houben R.M.G.J., Glynn J.R., Corbett E.L., Kranzer K. (2014). Pre-treatment loss to follow-up in tuberculosis patients in low- and lower-middle-income countries and high-burden countries: A systematic review and meta-analysis. Bull. World Health Org..

[5] Pai M., Clouse K., Blevins M., Lindegren M.L., Yotebieng M., Nguyen D.T., Omondi A., Michael D., Zannou D.M., Carriquiry G. (2017). Low implementation of Xpert MTB/RIF among HIV/TB co-infected adults in the International epidemiologic Databases to Evaluate AIDS (IeDEA) program. PLoS ONE.

[6] Theron G., Zijenah L., Chanda D., Clowes P., Rachow A., Lesosky M., Bara W., Mungofa S., Pai M., Hoelscher M. (2014). Feasibility, accuracy, and clinical effect of point-of-care Xpert MTB/RIF testing for tuberculosis in primary-care settings in Africa: A multicentre, randomised, controlled trial. Lancet.

[7] Cox H., Dickson-Hall L., Ndjeka N., Van’t Hoog A., Grant A., Cobelens F., Stevens W., Nicol M. (2017). Delays and loss to follow-up before treatment of drug-resistant tuberculosis following implementation of Xpert MTB/RIF in South Africa: A retrospective cohort study. PLoS Med..

[8] Cambanis A., Ramsay A., Wirkom V., Tata E., Cuevas L.E. (2007). Investing time in microscopy: An opportunity to optimise smear-based case detection of tuberculosis. Int. J. Tuberc. Lung Dis..

[9] Hanscheid T. (2008). The future looks bright: Low-cost fluorescent microscopes for detection of *Mycobacterium tuberculosis* and Coccidiae. Trans. R. Soc. Trop. Med. Hyg..

[10] Ristenpart W.D., Kim P.G., Domingues C., Wan J., Stone H.A. (2007). Influence of substrate conductivity on circulation reversal in evaporating drops. Phys. Rev. Lett..

[11] Trantum J.R., Baglia M.L., Eagleton Z.E., Mernaugh R.L., Haselton F.R. (2014). Biosensor design based on Marangoni flow in an evaporating drop. Lab Chip.

[12] Trantum J.R., Eagleton Z.E., Patil C.A., Tucker-Schwartz J.M., Baglia M.L., Skala M.C., Haselton F.R. (2013). Cross-sectional tracking of particle motion in evaporating drops: Flow fields and interfacial accumulation. Langmuir.

[13] Trantum J.R., Wright D.W., Haselton F.R. (2012). Biomarker-mediated disruption of coffee-ring formation as a low resource diagnostic indicator. Langmuir.

[14] Magana Vergara C., Kallenberg C.J.L., Rogasch M., Hubner C.G., Song Y.H. (2017). A versatile vector for mycobacterial protein production with a functional minimized acetamidase regulon. Protein Sci..

[15] Goude R., Parish T., Parish T., Brown A.C., Walker J.M. (2008). Electroporation of Mycobacteria. Mycobacteria Protocols.

[16] Shea T.M. (2008). Durable Hydrophobic Surface Coatings Using Silicone Resins. U.S. Patent.

[17] Shea T.M. (2005). Durable Hydrophobic Surface Coatings Using Silicone Resins. U.S. Patent.

[18] Jamieson D.T., Irving J.B., Klemens P.G., Chu T.K. (1976). Thermal Conductivity of Silicone Oils of the Polymethylphenyl Siloxane Type. Thermal Conductivity 14.

[19] Ishkhanov A.M., Nemzer V.G., Pugach V.V., Rastorguev Y.L. (1975). Thermal Conductivity of Polymethylphenylsiloxanes at High Pressures. J. Eng. Phys..

[20] (2017). SYLGARD 184 Silicone Elastomer—Technical Data Sheet.

[21] (1996). Teflon PTFE Fluoropolymer Resin—Properties Handbook.

[22] Touloukian Y.S., Powell R.W., Ho C.Y., Klemens P.G. (1970). Thermal Conductivity—Nonmetalic Solids.

[23] Thuau D., Koymen I., Cheung R. (2011). A microstructure for thermal conductivity measurement of conductive thin films. Microelectron. Eng..

[24] Huang D., Swanson E.A., Lin C.P., Schuman J.S., Stinson W.G., Chang W., Hee M.R., Flotte T., Gregory K., Puliafito C.A. (1991). Optical coherence tomography. Science.

[25] Chedore P., Th’ng C., Nolan D.H., Churchwell G.M., Sieffert D.E., Hale Y.M., Jamieson F. (2002). Method for inactivating and fixing unstained smear preparations of *Mycobacterium tuberculosis* for improved laboratory safety. J. Clin. Microbiol..

[26] Schneider C.A., Rasband W.S., Eliceiri K.W. (2012). NIH Image to ImageJ: 25 years of image analysis. Nat. Methods.

[27] Van Deun A., Aung K.J., Khan M.H., de Jong B.C., Gumusboga M., Hossain M.A. (2014). An operational study comparing microscopes and staining variations for tuberculosis LED FM. Int. J. Tuberc. Lung Dis..

[28] Lumb R., Van Deun A., Bastian I., Fitz-Gerald M. (2013). Laboratory Diagnosis of Tuberculosis by Sputum Microscopy—The Handbook, Global Edition.

[29] Deegan R.D., Bakajin O., Dupont T.F., Huber G., Nagel S.R., Witten T.A. (1997). Capillary flow as the cause of ring stains from dried liquid drops. Nature.

[30] Deegan R.D., Bakajin O., Dupont T.F., Huber G., Nagel S.R., Witten T.A. (2000). Contact line deposits in an evporating droplet. Phys. Rev. E.

[31] Deegan R.D. (2000). Pattern formation in drying droplets. Phys. Rev. E.

[32] Hu H., Larson R.G. (2005). Analysis of the Microfluidic Flow in an Evaporating Sessile Droplet. Langmuir.

[33] Hu H., Larson R.G. (2005). Analysis of the Effects of Marangoni Stresses on the Microflow in an Evaporating Sessile Droplet. Langmuir.

[34] Barash L.Y., Bigioni T.P., Vinokur V.M., Shchur L.N. (2009). Evaporation and fluid dynamics of a sessile drop of capillary size. Phys. Rev. E.

[35] Brutin D., Sobac B., Loquet B., Sampol J. (2010). Pattern formation in drying drops of blood. J. Fluid Mech..

[36] Rathaur V.S., Kumar S., Panigrahi P.K., Panda S. (2020). Investigating the Effect of Antibody-Antigen Reactions on the Internal Convection in a Sessile Droplet via Microparticle Image Velocimetry and DLVO Analysis. Langmuir.

[37] Stetten A.Z., Iasella S.V., Corcoran T.E., Garoff S., Przybycien T.M., Tilton R.D. (2018). Surfactant-induced Marangoni transport of lipids and therapeutics within the lung. Curr. Opin. Colloid Interface Sci..

[38] Yano Y.F., Ina T., Uruga T. (2021). Understanding the Dynamics of a Lipid Monolayer on a Water Surface under a Marangoni Flow. Colloids Interfaces.

[39] Trantidou T., Elani Y., Parsons E., Ces O. (2017). Hydrophilic surface modification of PDMS for droplet microfluidics using a simple, quick, and robust method via PVA deposition. Microsyst.

[40] Spitze L.A., Richards D.O. (1947). Surface Studies of Glass. Part I. Contact Angles. J. Appl. Phys..

[41] Sumner A.L., Menke E.J., Dubowski Y., Newberg J.T., Penner R.M., Hemminger J.C., Wingen L.M., Brauers T., Finlayson-Pitts B.J. (2004). The nature of water on surfaces of laboratory systems and implications for heterogeneous chemistry in the troposphere. Phys. Chem. Chem. Phys..

[42] Schatz M.F., Neitzel G.P. (2001). Experiments on Thermocapillary Instability. Annu. Rev. Fluid. Mech..

[43] Hobby G.L., Holman A.P., Iseman M.D., Jones J.M. (1973). Enumeration of tubercle bacilli in sputum of patients with pulmonary tuberculosis. Antimicrob. Agents Chemother..

[44] Lewis J.J., Chihota V.N., van der Meulen M., Fourie P.B., Fielding K.L., Grant A.D., Dorman S.E., Churchyard G.J. (2012). “Proof-of-concept” evaluation of an automated sputum smear microscopy system for tuberculosis diagnosis. PLoS ONE.

[45] Steingart K.R., Ng V., Henry M., Hopewell P.C., Ramsay A., Cunningham J., Urbanczik R., Perkins M.D., Aziz M.A., Pai M. (2006). Sputum processing methods to improve the sensitivity of smear microscopy for tuberculosis: A systematic review. Lancet Infect. Dis..

[46] Steingart K.R., Henry M., Ng V., Hopewell P.C., Ramsay A., Cunningham J., Urbanczik R., Perkins M., Aziz M.A., Pai M. (2006). Fluorescence versus conventional sputum smear microscopy for tuberculosis: A systematic review. Lancet Infect. Dis..

[47] Ghodbane R., Drancourt M. (2013). Magnetic bead protocol for culturing *Mycobacterium tuberculosis* from sputum specimens. J. Clin. Micro..

[48] Peterson E.M., Nakasone A., Platon-DeLeon J.M., Jang Y., de la Maza L., Desmond E. (1999). Comparison of Direct and Concentrated Acid-Fast Smears to Identify Specimens Culture Positive for *Mycobacterium* spp.. J. Clin. Microbiol..

[49] Albert H., Ademun P.J., Lukyamuzi G., Nyesiga B., Manabe Y., Joloba M., Wilson S., Perkins M.D. (2011). Feasibility of magnetic bead technology for concentration of mycobacteria in sputum prior to fluorescence microscopy. BMC Infect. Dis..

[50] Wilson S., Lane A., Rosedale R., Stanley C. (2010). Concentration of *Mycobacterium tuberculosis* from sputum using ligand-coated magnetic beads. Int. J. Tuberc. Lung Dis..

[51] Sahile H.A., Rens C., Shapira T., Andersen R.J., Av-Gay Y. (2020). DMN-Tre Labeling for Detection and High-Content Screening of Compounds against Intracellular Mycobacteria. ACS Omega.

[52] Hu H., Larson R.G. (2006). Marangoni Effect Reverses Coffee-Ring Depositions. J. Phys. Chem. B.

[53] Still T., Yunker P.J., Yodh A.G. (2012). Surfactant-induced Marangoni eddies alter the coffee-rings of evaporating colloidal drops. Langmuir.

[54] Jeon K., Koh W.-J., Kwon O.J., Suh G.Y., Kim H., Lee N.Y., Park Y.K., Bai G.H. (2005). Recovery rate of NTM from AFB smear-positive sputum speciments at a medical centre in South Korea. Int. J. Tuberc. Lung Dis..

[55] Pearlman S.I., Leelawong M., Richardson K.A., Adams N.M., Russ P.K., Pask M.E., Wolfe A.E., Wessely C., Haselton F.R. (2020). Low Resource Nucleic Acid Extraction Method Enabled by High-Gradient Magnetic Separation. ACS Appl. Mater. Interfaces.

[56] Miltenyi S., Muller W., Weichel W., Radbruch A. (1990). High Gradient Magnetic Cell Separation with MACS. Cytometry.

[57] Yoon S.H., Lee N.K., Yim J.J. (2012). Impact of sputum gross appearance and volume on smear positivity of pulmonary tuberculosis: A prospective cohort study. BMC Infect. Dis..

[58] Salehi-Reyhani A., Sharma S., Burgin E., Barclay M., Cass A., Neil M.A., Ces O., Willison K.R., Klug D.R., Brown A. (2013). Scaling advantages and constraints in miniaturized capture assays for single cell protein analysis. Lab Chip.

