# Supplementary material for: Controlling Droplet Marangoni Flows to Improve Microscopy-Based TB Diagnosis
Source: Diagnostics (Basel). 2021 Nov 21;11(11):2155. doi: 10.3390/diagnostics11112155 (PMC8618690; doi:10.3390/diagnostics11112155)
Supplement: Supplementary file 1 [file diagnostics-11-02155-s001.zip › Supplementary Figures ver F.pdf]

SUPPORTING INFORMATION

Controlling droplet Marangoni flows to improve microscopy-based TB diagnosis

Stephanie I. Pearlman, Eric M. Tang, Yuankai K. Tao, Frederick R. Haselton*

Department of Biomedical Engineering, Vanderbilt University, Nashville, TN

*CORRESPONDING AUTHOR: rick.haselton@vanderbilt.edu

SUPPLEMENTARY VIDEO CAPTIONS

Video S1. Sequential OCT images of evaporating 1 µL droplet containing 10^6 polystyrene particles/uL, which are 1 µm in diameter. A total of 800 repeated frames were taken at the same position for a total acquisition time of 36 seconds per measurement in order to monitor the changes in particle flow and the droplet evaporation. For each droplet, measurements were taken at two minute intervals beginning at one minute following droplet placement until complete evaporation.

Video S2. Sequential fluorescence microscopy images at 100x total magnification of evaporating 5 µL droplet containing 10^4 polystyrene particles/uL, which are 1 µm in diameter. A total of 40 frames at 100 ms exposure were taken at the same position near the solvent-substrate for a total acquisition time of 40 seconds per measurement in order to monitor the changes in particle flow and the droplet evaporation. In focus particles move inward along the substrate. Once particles reach the center of the droplet, they become out of focus, and begin moving in the opposite direction. This is consistent with the particles moving upward towards the top of the droplet, then moving outward along the outer surface of the droplet. Combined, the particle movement is consistent with the Marangoni flow pattern shown in **Figure 1**.

SUPPLEMENTARY FIGURES

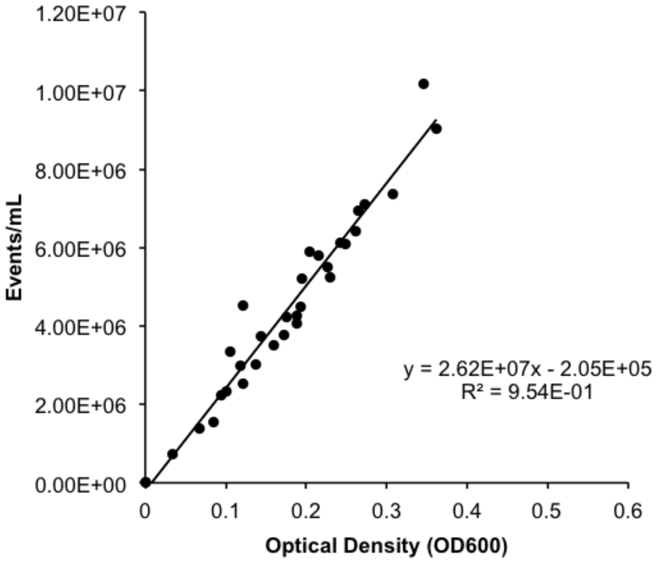

Figure S1. Flow cytometry curve correlating number of events counted against OD600 of *M. bovis* BCG bacteria.

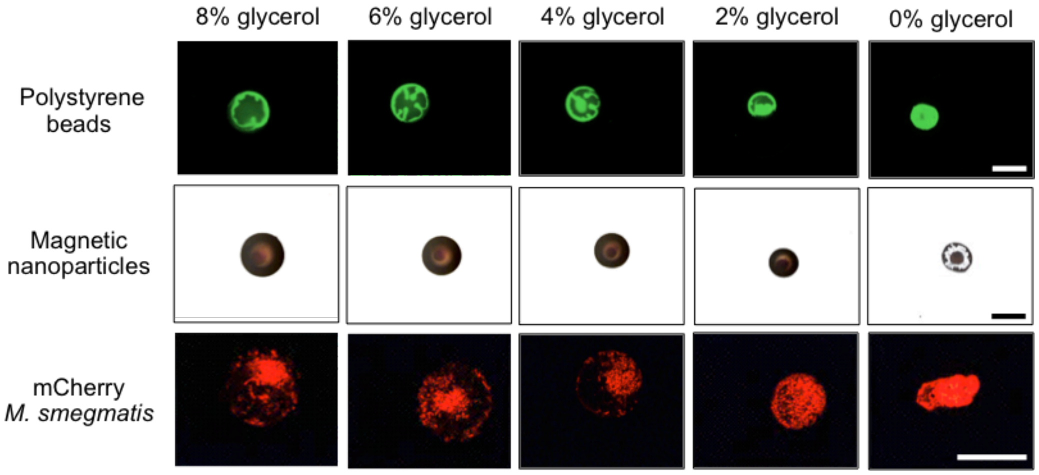

Figure S2. Removing glycerol from droplet solvent did not significantly change particle deposition location in droplets containing 1 µm polystyrene beads (top), 200 nm iron oxide magnetic nanoparticles (middle), or mCherry expressing *M. smegmatis* bacteria (bottom). Scale bars = 500 µm.

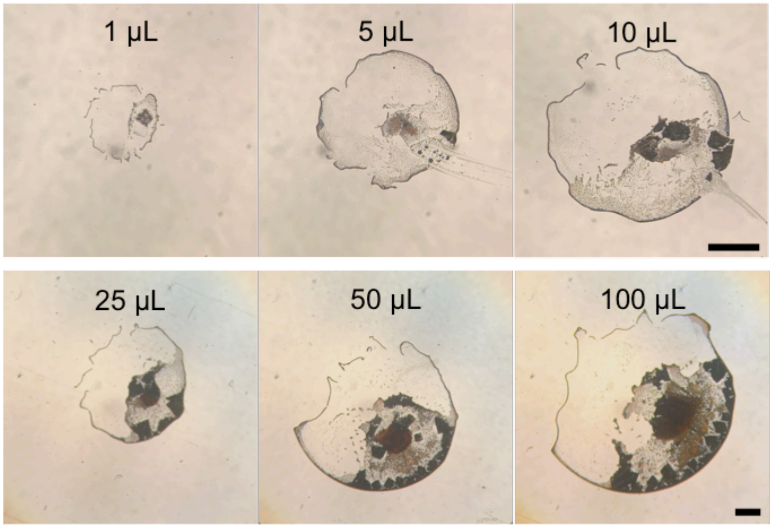

Figure S3. The size of the droplet did not impact the final deposition pattern of the droplet, up to a 100 μL volume. All droplets contained nanoparticles at a concentration of 0.2 mg/mL. Ambient temperature was 23°C. Images were taken on Nikon TE-200U with an iPhone and 10x smartphone camera adapter. The image temperature was adjusted for publication to reduce the orange hue of the image. Scale bars = 1 mm.

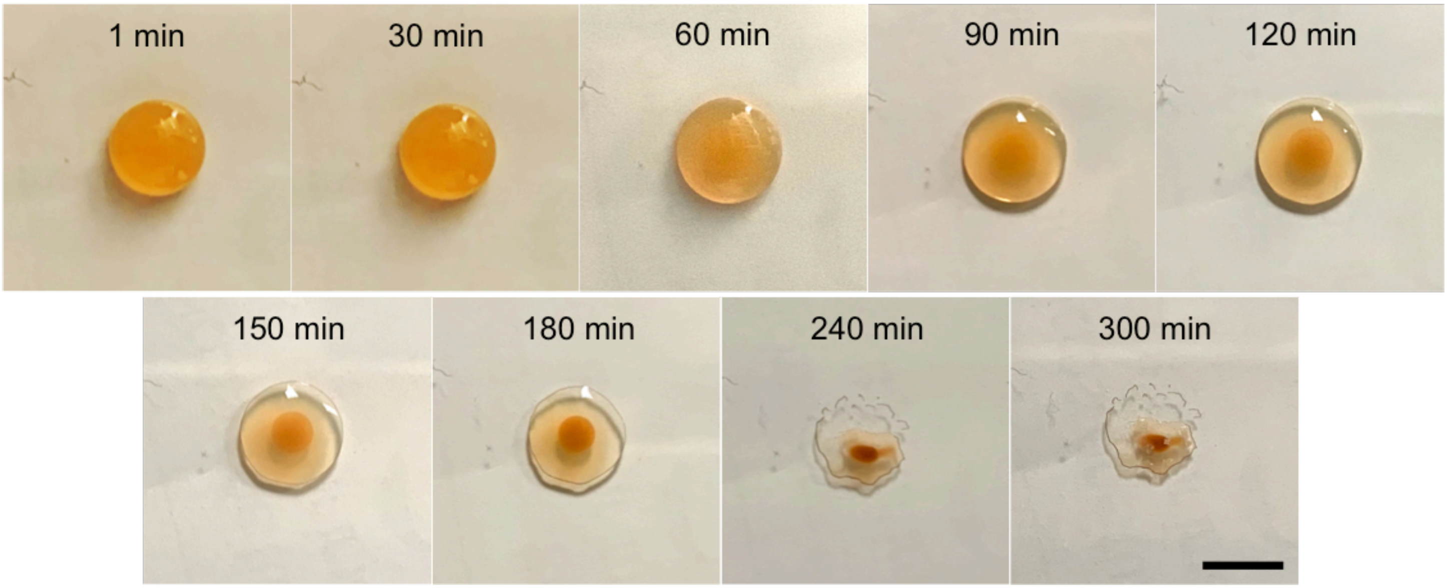

Figure S4. Time-lapse imaging of a 50 μL evaporating droplet shows the formation of the central deposition area as the droplet evaporated. Images were captured ever 30 min for the first three hours, and once an hour thereafter until evaporation was complete after 5 hours. Droplet contained nanoparticles at 0.2 mg/mL. Ambient temperature was 20°C. The shadows of the centrally concentrated nanoparticles and edges of the droplet observed are reflections off the backside of the glass slide. Images taken with an iPhone. Scale bar = 5 mm.

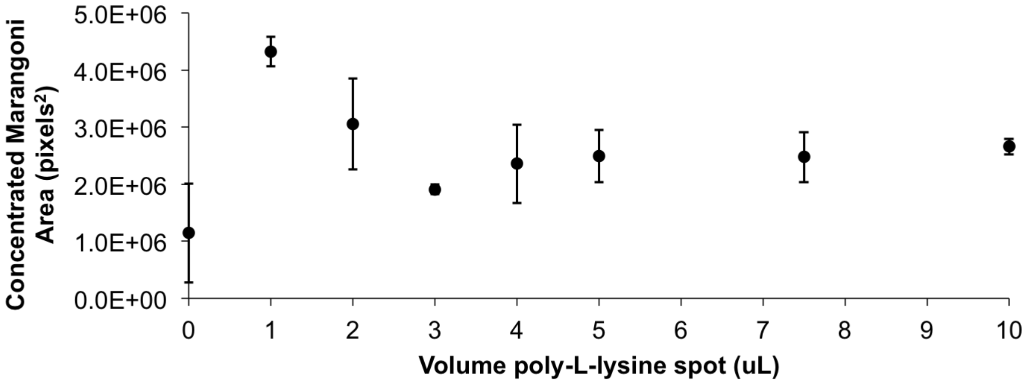

Figure S5. Effect of poly-L-lysine landing zone size on area of central Marangoni deposition area (mean ± s.d., n ≥ 6).

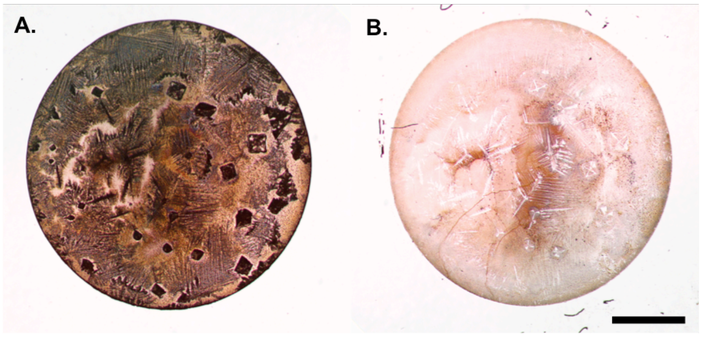

Figure S6. Drying of 50 μL droplet on plain glass did not result in characteristic Marangoni deposition pattern upon complete drying. **A.** Before rinsing off salt crystals. **B.** After rinsing off salt crystals. Droplet was imaged using Nikon TE 200-U using a Nikon DS-Ri1 camera at 10x total magnification since the entire droplet could not be imaged in a single field of view using optical setup on the Olympus CX23. To reduce the effects of uneven illumination using ImageJ [19], nanoparticle droplets had a representative background image subtracted from the original deposition image. Scale bar = 1 mm.
